# Improving Family Medicine Residents’ Written Communication Using a Self-assessment Process

**Published:** 2012-03-31

**Authors:** José François

**Affiliations:** University of Manitoba, Winnipeg, Manitoba, Canada

## Abstract

**Background:**

Although competency in written communication is a core skill, written communication is seldom the focus of formal instruction in medical education. The objective of this intervention was to implement a self-assessment strategy to assist learners in improving their letter writing skills and then to evaluate its feasibility, reliability and potential educational value.

**Methods:**

Eight first-year family medicine residents from two teaching sites completing a six month family medicine rotation used a self-assessment process which included a self-study module and an assessment tool for letters. Each resident applied the self-assessment tool to eight to ten consecutive consult/referral request letters. Participants submitted initial and redrafted letters for independent rating.

**Results:**

Analysis of the content, style and global ratings of the initial 77 draft letters showed multiple deficiencies in the content of their letters. It was confirmed that by using the self-assessment tool, residents were able to reliably assess the quality of their letters. Residents’ assessments and those of the expert closely correlated (Pearson correlation 0.861, *p* < 0.0001). Over the course of the study the residents’ overall performance improved and the difference in total scores between the initial drafts and the rewritten letters narrowed.

**Conclusion:**

A self-assessment process of written communication significantly improves the quality and completeness of routine consult/referral request letters.

## Background

As an increasing amount of patient care is occurring in the outpatient setting, written communication, in the form of consult/referral request and reply letters, has become the most common means by which doctors exchange information pertinent to patient care.^1^,^2^,^3^ It is surprising that, despite its importance to practice, surveys of communication skills programs show that written communication is seldom the focus of formal instruction in medical education.^4^,^5^,^6^ Medical schools have acknowledged a need for writing instruction in their curricula but have identified several barriers to its inclusion, including time constraints in a crowded curriculum, lack of interest and lack of qualified staff to teach such courses.^4^,^6^,^7^

An independent learning approach to letter writing using a self-study module and a self-assessment tool was conceived as it would be an efficient, cost-effective and pedagogically appropriate approach with the additional benefit of not having to compete for time in an already busy curriculum. Traditional teaching methods generally do not emphasize self-learning skills, although there is growing recognition that they are important to becoming effective lifelong learners.^8^

## Methods

Eleven first-year family medicine residents starting a six-month family medicine rotation at two sites of the University of Manitoba’s Family Medicine Residency training program were invited to participate. Eight of eleven eligible residents consented to participate in the project.

Each participant received a file-folder which included ten copies of a previously described assessment tool for letters as well as a self-study module on best practices in written communication in medicine.^9^ A systematic review of literature on the topic of evaluation tools for specialty residents’ letters had provided the background for the design of an asessment tool for consult and referral request letters.^10^–^13^ The items for the assessment tool were generated from audits of consultation/referral request letters and surveys of recipient specialists highlighting the necessary content of letters.^1^,^14^–^19^

Participants were instructed to complete the self-study module and then use the letter assessment tool after each of ten consecutive consult/referral request letters they dictated. After applying the assessment tool (self-assessment), the participants were asked to correct or redraft their letter to improve its quality and completeness based on the deficiencies they may have identified when using the tool. Residents were instructed to return to the file folder copies of the initially drafted letter (**Pre-**), the completed self-assessment tool, and the re-drafted letter (**Post-**).

Pre- and post-letters were collected and then forwarded for independent rating to one of two recruited family physician teachers who were not members of either of the teaching units. The assessment tool scored by the participant (self-assessed score) for each letter was also collected for correlation with the independent rater’s scores. To ensure anonymity and confidentiality, all patient and resident physician identifiers were removed prior to being sent for rating.

## Results

A total of 77 letters from the 8 participating residents were collected (2 residents submitted only 9 letters for review and 1 resident’s letter was rejected as it was not a consult/referral request letter). The analysis of individual content, style and global ratings of residents’ first few attempts in composing consult/referral request letters shows multiple deficiencies (Table 1). When analyzing the first 3 letters submitted by participants, close to one-third (29.2%) of letters had no clearly defined statement regarding the reason for the referral, one out of four (25.0%) did not provide a history of previous medical problems and almost half (45.8%) did not provide a previous surgical history. Over one-third of letters (37.5%) did not provide relevant psychosocial history. Updated medication lists were absent one-third of the time (37.5%) and few (29.8%) of the residents’ first letters provided any information regarding the presence or absence of allergies. In terms of writing style, initial letters frequently used long paragraphs (45.8% of letters had paragraphs longer than 5 lines) and almost half (45.8%) had more than one topic per paragraph.

As expected, the redrafted (post-) letters were consistently better than initial attempts (pre-), with some instances where all of the post-letters contained the desired information. The global assessment scores, out of 5 points, also increased between the pre- (3.88 ± 1.09) and post-letters (4.60± 0.59), suggesting an overall improvement of quality after using the tool. In addition, the global assessment score attributed by the independent rater also appears to correlate with the sum of all items thus providing a reliable scale (*F* value 5.93, *p* < 0.0001).

**Figure 1:**
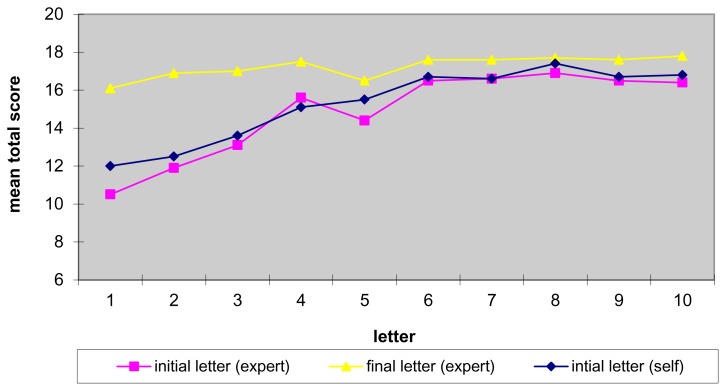
Mean total scores by letter number (n = 77)

When tracking resident performance over subsequent letters, it was observed that residents’ initial performance (Pre**-**letters), as exemplified by the total number of items, improves. On letter number 1, residents had, on average, only 12 of 18 items and by their final letter, this had increased to 16.8 items out of 18 (Figure 1). As expected, the redrafted letters are consistently better than the initial drafts and, as performance improves, the difference in total score between the initial draft and the redrafted letter narrows. This improvement is most rapid over the first 4 letters and appears to plateau at about the 6^th^ letter. Even after performance plateaus, it appears that redrafting with the use of the tool continues to make letters better.

The tool’s ability to assist residents in correctly judging their performance was assessed by comparing the independent rater’s scores with the scores on the self-assessment. Using Pearson correlation coefficients, it was confirmed that the residents’ assessment and those of the expert closely correlate (Pearson correlation 0.86059, *p* < 0.0001).

## Discussion

The approach utilized is different from previously reported interventions focusing on improving the quality and completeness of physicians’ letters in two respects: 1) it utilizes a self-study module for teaching on the ideal content and format of consult/referral request letters; and 2) it utilizes a self-assessment instrument. Traditional teaching methods in medical education generally do not emphasize self-learning or self-assessment skills, possibly due to the mixed results of past research.

The self-study module provided residents with an overview of the consultation and referral process as well as the necessary content and style elements of letters. The module provides benchmarking, which can increase the accuracy of self-assessment by increasing learners’ awareness of the standards to be achieved.^20^–^21^

The study was able to assess the accuracy of the self-assessment by comparing the total scores as determined by the resident and by the expert. Although the total scores as reported by residents and experts correlate, the use of dichotomous (YES/NO) items may not sufficiently differentiate in terms of quality as they are ‘all or none’. The use of dichotomous items rather than scales in the construction of this tool was deliberate as it was thought that the use of scales would be more difficult for novices as they would have relatively few comparison points that would allow them to discriminate between a poor, a good, a very good or an excellent letter. The results do show us that residents are able to accurately identify the presence or absence of specific content and style items in their letters.

An independent learning approach to teaching letter writing skills using a self-study module appears to be an efficient, cost-effective and pedagogically appropriate approach to teaching of written communication. This form of “just-in-time” education has been well received by both residents and teaching staff and did not require changes in the lecture schedule of the residency teaching program. The self-assessment process integrated itself well to the ambulatory teaching setting, did not increase the workload of teachers and had only a mild impact on resident workload. Myers et al.^11^ have commented that, in their experience, when given the opportunity to edit their letters, residents make only minor changes. This study shows that the addition of a self-assessment tool prompts residents to do more extensive redrafting of their letters than they would have otherwise done. This would suggest that the use of a self-assessment tool provides residents with immediate feedback and an opportunity to reflect on an essential skill for medical practice.

## Conclusion

This project demonstrates that learning and assessment of consult/referral request letter writing can be effectively and feasibly taught using the proposed approach. Teaching residents the constituent elements of consultation letters and having them reflect on their work not only improves the quality of written letters, it likely promotes a deeper understanding of the consultation and referral processes.

## Figures and Tables

**Table 1:** Presence (in % of letters) of content and style items in resident’s letters & global score of letters as determined by the independent rater.

	First 3 letters (n = 24)	Total number of letters (n = 77)	
	Pre-	Pre-	Post-
**A.** **Content** (dichotomous items)			
1) Patient demographics:	100.0%	100.0%	100.0%
2) Initial statement identifying the reason for the referral	70.8%	85.7%	100.0%
3) Description of chief complaint	91.7%	94.8%	97.4%
4) Description of associated symptoms	66.7%	85.7%	94.8%
5) Description of relevant collateral history	83.4%	87.0%	94.8%
6) Past medical history	75.0%	87.0%	100.0%
7) Past surgical history	54.2%	74.0%	89.6%
8) Relevant psycho-social history	62.5%	76.6%	89.6%
9) Current medication list	62.5%	83.1%	100.0%
10) Allergies	29.8%	66.2%	94.8%
11) Relevant clinical findings	62.5%	77.9%	92.2%
12) Results of investigations to date	75.0%	77.9%	92.2%
13) Outline of management to date	41.6%	76.6%	85.7%
14) Provisional diagnosis/clinical impression	70.8%	77.9%	97.4%
15) Statement of what is expected from the referral	83.4%	93.5%	100.0%

**B.** **Style** (dichotomous items)			
16) One topic per paragraph	45.8%	72.7%	98.7%
17) Paragraphs with fewer than 5 sentences	45.8%	74.0%	94.8%
18) One idea per sentence	79.2%	87.0%	98.0%

**C.** **Global** (scaled items, 1–5)			
19) Global assessment	2.96 (± 0.92)	3.88 (± 1.09)	4.60 (± 0.59)
